# “Mapping suicide prevention initiatives targeting Indigenous Sámi in Nordic countries”

**DOI:** 10.1186/s12889-021-12111-x

**Published:** 2021-11-07

**Authors:** Jon Petter A. Stoor, Heidi A. Eriksen, Anne C. Silviken

**Affiliations:** 1 Department of Epidemiology and Global Health, Umeå University, SE-901 87 Umeå, Sweden; 2 Centre for Sámi Health Research, Department of Community Medicine, UiT – the Arctic University of Norway, Tromsø, Norway; 3 Utsjok Primary Health Care Centre, Utsjok, Finland; 4 Sámi Norwegian National Advisory Unit for Mental Health and Substance Use, Finnmark Hospital Trust, Karasjok, Norway

**Keywords:** Sami, Saami, Indigenous, Arctic, Suicide prevention, Mental health, WPR-approach

## Abstract

**Background:**

Suicide is a major public health issue among Indigenous Sámi in Nordic countries, and efforts to prevent suicide in the Sámi context are increasing. However, there is no literature on suicide prevention initiatives among Sámi. The aim of the study was to map suicide prevention initiatives targeting Sámi in Norway, Sweden, and Finland during 2005–2019.

**Method:**

Initiatives were identified and described through utilizing networks among stakeholders in the field of suicide prevention among Sámi, acquiring documentation of initiatives and utilizing the authors first-hand experiences. The described initiatives were analyzed inspired by the “What is the problem represented to be?” (WPR)-approach.

**Results:**

Seventeen initiatives targeting Sámi were identified during 2005–2019, including nine in Sweden, five in Norway, one in Finland and two international initiatives. Analysis with the WPR-approach yielded 40 problematizations regarding how to prevent suicide among Sámi, pertaining to shortcomings on individual (5), relational (15), community/cultural (3), societal (14) and health systems levels (3). All initiatives were adapted to the Sámi context, varying from tailor-made, culture-specific approaches to targeting Sámi with universal approaches. The most common approaches were the gatekeeper and mental health literacy training programs. The initiatives generally lacked thorough evaluation components.

**Conclusion:**

We argue that the dominant rationales for suicide prevention were addressing shortcomings on individual and relational levels, and raising awareness in the general public. This threatens obscuring other, critical, approaches, such as broadening perspectives in prevention planning, improving health systems for Sámi, and promoting cultural empowerment among Sámi. Nevertheless, the study confirms considerable efforts have been invested into suicide prevention among Sámi during the last 15 years, and future initiatives might include a broader set of prevention rationales. To improve evaluation and identify the most promising practices, increased support regarding development of plans and implementation of evaluation components is needed.

**Supplementary Information:**

The online version contains supplementary material available at 10.1186/s12889-021-12111-x.

## Background

Suicide is a public health issue of much concern in the Arctic, especially among the Indigenous peoples due to their higher suicide mortality compared to the non-Indigenous peoples [1]. Among Sámi, the Indigenous people of northern Norway, Sweden, Finland, as well as the Kola Peninsula in Russia, the situation has been better than the Indigenous peoples elsewhere in the region. However, cohort studies highlighting suicide rates for Sámi in northern Norway (1970–1998), Sweden (1961–2000) and Finland (1979–2010) [1–4] show that suicide rates have been consistently elevated among Sámi men compared to non-Sámi men. Although the suicide rates among Sámi in Nordic countries (not including Sámi in northwestern Russia, for whom no data are available) are lower, suicide among Sámi in Norway and among reindeer herding Sámi in Sweden still share some characteristics with suicide in other Indigenous Arctic groups, including the commonality of violent methods and that young men are most vulnerable [1, 3, 5].

The World Health Organization (WHO) maintains that suicide is a multifaceted public health problem that calls for countries to strategize, coordinate and implement complex and comprehensive prevention plans. Preventive actions can be universal (target the whole population); focus on at-risk groups, such as minority groups with elevated rates of suicide; or at-risk individuals, such as people exhibiting suicidal behavior [6]. The WHO highlights that Indigenous peoples often qualify as at-risk groups, and suicide in those contexts should be understood in relation to community and group factors related to the colonization of Indigenous territories: “among Indigenous groups, territorial, political and economic autonomy are often infringed and native culture and language negated. These circumstances can generate feelings of depression, isolation and discrimination, accompanied by resentment and mistrust of state-affiliated social and health-care services, especially if these services are not delivered in culturally appropriate ways” [7]. However, influential scholars in the study of suicide prevention among Indigenous peoples in the US have critiqued previous research in the field for being almost exclusively focusing on “universally measurable individual variables” [8]. They suggest that, although such approaches have revealed much individual despair among Indigenous peoples, it has yielded few ways forward in terms of addressing the community-level causes of individual despair. Consequentially, current academic literature is increasingly focused on understanding above-individual-level factors (e.g., family, community, social, cultural, contextual, political and historical factors), as well as exploring strength-based, community grounded prevention approaches [8, 9], also in the Arctic [10–12]. Furthermore, Indigenous peoples are themselves adding to such perspectives through developing their own suicide prevention policies, for example, in Australia and northern Canada [13, 14].

Finland was the first country in the world to start a national suicide prevention program in the late 1980s [6]. However, neither that program nor subsequent national plans in Nordic parts of Sápmi (Norway, Sweden and Finland) have included Sámi in meaningful ways. For example, the 2014–2017 national action plan to prevent suicide and self-harm in Norway [15] mentioned Sámi briefly, but did not articulate any aim or action targeting them. In Sweden, the current national prevention strategy does not mention Sámi at all, although it aims at reducing suicide to zero, supposedly indicating its high political priority [16]. The lack of inclusion of Sámi in the national prevention policies was the reason for creating the “Plan for Suicide Prevention among the Sámi people in Norway, Sweden and Finland” (PSPS) [17], developed through a joint effort between a regional branch of the Norwegian health system (tasked with ensuring equity in mental health outcomes for Sámi in Norway) and the Saami Council, a pan-Sámi non-governmental organization (NGO).

The eleven strategies included in the PSPS (see Table 1) are meant to be complementary to the existing universal prevention approaches. They build both on the perspectives of Sámi “grassroots” of suicide prevention (included through workshops) and the limited available scientific evidence concerning suicide among Sámi. Notably, some of the strategies (e.g. strategy 3, 4 and 5) may be interpreted as reflecting a similar development to that described in the US [8], with the importance of socio-political factors being highlighted. This is also in adherence with recent qualitative studies suggesting that Sámi themselves understand suicide among them as related to contextual factors like loss of Indigenous identity, language, culture and land rights [18, 19]. However, there has been at least two failed attempts at identifying literature on suicide prevention initiatives targeting Sámi [11, 20]. This means that neither knowledge regarding what suicide prevention projects have been carried out, the reach, and outcomes of those projects nor their prevention rationales, is available in the Sámi context. Therefore, the aim of this study was to identify, describe and analyze suicide prevention initiatives targeting Sámi in Norway, Sweden, and Finland during 2005–2019.

**Table 1:** The eleven prevention strategies included in the “Plan for Suicide Prevention among the Sámi people in Norway, Sweden and Finland” [17]

1. Focusing efforts on the Sámi men	
2. Producing statistics and strengthening research on suicide among the Sámi	
3. Strengthening Sámi self-determination	
4. Initiating efforts to recognise and deal with historical traumas	
5. Strengthening and protecting the Sámi cultural identity	
6. Reducing the Sámi’s exposure to violence	
7. Reducing the Sámi’s experiences of ethnic discrimination	
8. Increasing diversity and acceptance in the Sámi community	
9. Securing the Sámi’s right to equal, linguistically and culturally adapted mental health care	
10. Educating and mobilising the Sámi civil society for suicide prevention	
11. Initiating and strengthening cross-border cooperation for suicide prevention	

## Method

We (the authors) were not aware of any systematic replicable method for scoping the diverse sources that could potentially contain information regarding suicide prevention initiatives among Sámi in the Nordic countries. Because of this, we chose a pragmatic approach building on the particular strengths of our research group: our experiences from working in the field of suicide prevention among Sámi in Norway, Sweden and Finland, respectively. The mapping of initiatives was conducted in three steps: identifying, describing and analyzing. The purpose of the interpretative analysis was to clarify *how suicide prevention among Sámi was meant to function, as seen from the perspectives embedded within the identified prevention initiatives. This is important as suicide is a phenomenon characterized by its complexity; hence, there are many potential ways to seeks its prevention. Since those ways among Sámi have not been previously investigated in the academic context, it makes sense to provide a roadmap that both describes what has been done before and with what intentions. An adaptation of the* “What is the problem represented to be?” (WPR)-approach was selected as the analytical tool. The WPR-approach is a policy analysis tool developed by Carol Bacchi, which presupposes that *“what we propose to do about something indicates what we think needs to change and hence what we think the ‘problem’ is”* *[*21*]*. *It is a flexible method that can be employed in different ways (including parts of, or the whole original method), utilizing different data (written, verbal or non-verbal, including symbolic “languages”) for different purposes. Bacchi exemplified how the WPR-approach might work with an analysis of a policy regarding violence towards women, where the policy suggests that women should be given access to self-help courses. A WPR analysis of such a policy would then highlight how the policy “places” the problem of violence towards women (and the responsibility to fix it) among the women themselves, as opposed to elsewhere (among the perpetrators of violence, for example).*

### Identifying

Potential initiatives were identified through inquiring professionals, researchers, organizations and other stakeholders engaged in the field of suicide prevention among Sámi in Norway, Sweden and Finland. As there is no coordination or system available for tracking suicide prevention initiatives in this context, the author’s extensive networks within respective countries was helpful in this process, which was carried out by phone and email. The timeframe was limited to initiatives that had taken place in the period 2005–2019 because this was the timeframe during which the authors had most experience from working in the field of suicide prevention among Sámi. A list of initiatives was compiled, and organizations/institutions responsible for administering and/or funding initiatives were requested to provide project reports and other documentation regarding those initiatives. The list included initiatives explicitly aimed at preventing suicide among Sámi, as well as some initiatives without this being explicit. The latter were included if other available information suggested that the initiative was relevant, although not explicitly framed as suicide prevention because of a need to conform to funding mechanisms primarily meant to support other goals. For example, this allowed for inclusion of several prevention initiatives run by Sámi NGOs in Sweden that were funded by sources such as Swedish government grants geared towards supporting gender equity and youth engagement in civil society. Furthermore, universal suicide prevention initiatives in Sámi areas (i.e., not focusing explicitly on Sámi) were included if these included adaptations to fit Sámi needs, such as employing Sámi personnel for better reach among the Sámi population. To ensure sufficient amount of information regarding each included initiative; initiatives were excluded if no written documentation of the initiative was acquired while none of the co-authors had experience from working with the initiative (one initiative was excluded because of this).

**Describing**

A template, inspired by the review on suicide prevention projects in Aboriginal Australia by Ridani, Shand [22], was created and available information regarding the initiatives was inserted. Thus, the following descriptive data was included: name and year; aim or mission; target population and geographic reach (region, country); delivery method(s), including participant information (if available); administering organizations; funding institutions; and what evaluation data was reported. Information on what source(s) was used for compiling the descriptive data was also included.

### Analyzing

The compiled data was read through for the purpose of employing an interpretative analysis inspired by the WPR-approach *and combined with a thematical categorization of the problematizations found. In this case, the question “What is the problem(s) represented to be?” was posed to the descriptive data compiled in the template. The interpretation of how each initiative problematized suicide was thus based on the aims, delivery methods (activities carried out), target population, and other characteristics of the identified initiatives. In practice, this process was conducted through the first author making a preliminary suggestion as how to answer the question for each initiative, and the other authors checking, suggesting changes and/or confirming that those interpretations were reasonable, in a back and forth process (through emails and online meetings, due to physical distances). Several problematizations for each initiative were allowed, especially for initiatives with multiple prevention actions undertaken. The problematizations were then thematically categorized based on the content of the problematizations (where they placed the problem), informed by the WHO’s framework of suicide risk and protective factors as related to individual, relational, community/culture, social or health systems levels* [6, 7, 23]*.*

## Results

The sources used for compiling and describing the initiatives included 12 project reports concerning 11 initiatives, as well as first-hand accounts for 12 initiatives. The results are presented in the form of a summary of what problematizations of suicide were found to be embedded within the initiatives and what actions were carried out to address those problems. Also, a narrated overview of other characteristics of the identified initiatives was included. Detailed tables of identified initiatives (Supplementary Tables 1 and 2) and problematizations of suicide (Supplementary Table 3) are provided as supplementary material.

### Problematizations of suicide and actions to address those problems

**Fig. 1:**
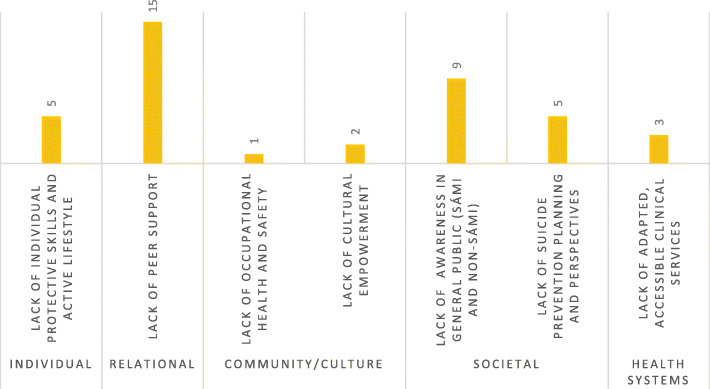
What are the problems represented to be in suicide prevention initiatives targeting Sámi? Number of problematizations in thematic categories and suggested corresponding levels of intervention

A total of 17 suicide prevention initiatives were identified to have targeted Sámi in Norway [5], Sweden [9], and Finland [1] specifically, as well as in all those countries internationally [2]. Analysis of the identified initiatives utilizing the “What is the problem represented to be?”-question yielded 40 problematizations regarding how to prevent suicide among Sámi. The problematizations were compared and thematically categorized depending on interpretation of where they “placed” the problem (i.e., what problems they aimed at fixing, as a way of preventing suicide) (Fig. 1). The thematic categories included “lack of individual protective skills and active lifestyle” (5 problematizations), “lack of peer support” [15], “lack of occupational health and safety” [1], “lack of cultural empowerment” [2], “lack of suicide prevention planning and perspectives” [5], “lack of awareness in general public (Sámi and non-Sámi)” [9] and “lack of adapted, accessible clinical services” [3].

Problematizations related to individual shortcomings included lack of active lifestyle, lack of coping skills, lack of conflict management skills, lack of tools for emotion regulation and lack of self-care skills among Sámi kids, youth and young adults. Activities to address these shortcomings included a local project providing a program for encouraging outdoors activities for at-risk youth. Furthermore, other projects arranged workshops focused on 1) exploring the use of yoiking as a tool for improved emotional regulation among young Sámi men (yoiking is a Sámi form of vocal cultural expression), 2) teaching conflict management tools and 3) promoting self-care skills for young male reindeer herders, and young reindeer herders (irrespective of sex), respectively. These workshops were based on in-group dialog rather than predefined curriculum.

The most common type of problematization (15 out of 40 problematizations) related suicide among Sámi to the relational level, specifically, “lack of peer support.” Activities meant to address this issue were aimed both at strengthening the general social support within specific groups of Sámi (such as youth and young adults, among men and/or within reindeer husbandry) and trying to achieve specific changes in the general Sámi population through implementation of mental health literacy and gate-keeper training programs. Almost all the activities aimed at addressing the relational level shortcomings were workshop-based (delivered in workshop formats), either with predefined curriculum from internationally used prevention programs, such as Applied Suicide Intervention Skills Training (ASIST) and Mental Health First Aid (MHFA), or using workshops to strengthen bonds and within-group support through talking about shared challenges. For the latter type of workshops, a common method was gathering youth/young adult Sámi and letting them listen to a slightly older peer, who functioned as a role model and shared his/her story of dealing with and overcoming suicidality, and then having the group talk and discuss the content recognized within that story. Another approach to increasing peer support was starting a telephone-based crisis hotline run by Sámi laypersons.

Problematizations of suicide related to the community and culture levels of intervention included both “lack of occupational health and safety” within reindeer herding and “lack of cultural empowerment” among Sámi youth and young Sámi men. Activities to address these problems included a project aimed at increasing and systematizing efforts to strengthen the occupational health and safety work within the reindeer herding industry and two workshop-based projects utilizing yoiking as tool for cultural empowerment among young Sámi.

Problematizations identified as related to the societal level of intervention included “lack of suicide awareness in both the Sámi and non-Sámi general populations” and “lack of suicide prevention planning and perspectives.” Activities aimed at increasing awareness of suicide included arranging open meetings to discuss suicide in the Sámi context, as well as producing information leaflets and reports containing information regarding the issue. Activities aimed at strengthening prevention planning and inclusion of relevant perspectives included, for example, creating prevention plans on a local community level and on a general Sámi level, as well as a project teaching Indigenous youth methods for digital storytelling in an effort to support their perspectives to be heard.

Lastly, problematizations related to health systems included “lack of adapted, accessible clinical services.” Activities aimed at addressing this included running a low-threshold psychiatric service focusing on suicidality and drug use among Sámi youth and young adults, trying to improve the general level of knowledge relating to Sámi health within the universal health care systems in northern Sweden (focusing on personnel in psychiatry and primary health care), as well as improving recognition and referral of at-risk youth from school-based nurses to other parts of the health system in a local community context.

### Characteristics of the identified initiatives

**Fig. 2:**
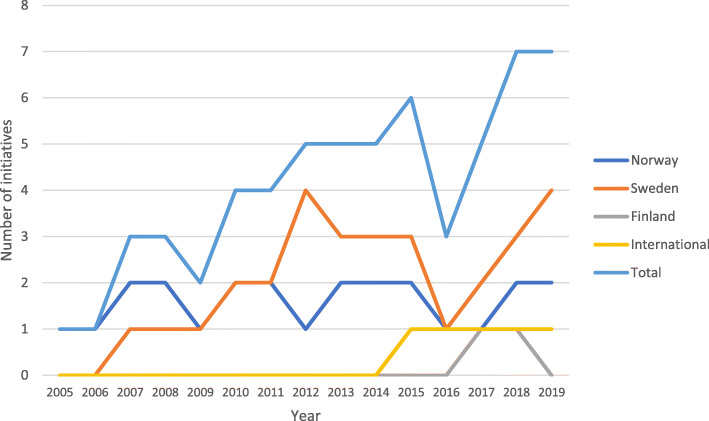
Number of suicide prevention initiatives targeting Sámi in Norway, Sweden, Finland and internationally, per year (2005–2019)

Only one initiative was identified for 2005–2006, but the number of initiatives rose thereafter (Fig. 2). From 2010 and onwards, about five initiatives have been simultaneously on-going. Four initiatives were still on-going and planned to continue during 2020, including three in Sweden and one in Norway.

Few initiatives connected their specific activities to overarching priorities of suicide prevention strategies. However, projects run by health care organizations were implicitly connected to national suicide prevention strategies, as their mission was to deliver prevention training workshops to Sámi, which were chosen for implementation on a national (universal) level. Also, one initiative referred to national prevention strategies, as it sought to create a Sámi-specific prevention plan meant to complement already existing universal plans. The initiatives targeted different groups, including the Sámi (and non-Sámi) general public, whole communities, youth, reindeer herders, young male reindeer herders, at-risk individuals (self-identified or identified by school personnel), and service providers.

In terms of delivery methods, 12 initiatives, at least to some degree, employed workshops and/or public meetings as delivery methods; eight of these included specific suicide prevention training workshops for improved mental health literacy and so-called gate-keeper training. These included two initiatives providing the 2-day Australian MHFA course among Sámi in Sweden, three initiatives providing the 2-day Canadian ASIST course among Sámi in Norway and one initiative providing it among Sámi in Finland. Furthermore, the half-day Canadian SafeTALK course was provided one time by one initiative in Sweden.

All initiatives were somewhat adapted to the Sámi context, although the width of adaptations was wide, ranging from the only adaptation being to recruit Sámi participants for an otherwise not adapted prevention activity to creating tailor-made approaches for specific Sámi groups, utilizing Sámi cultural activities as part of the initiative. The initiatives implementing ASIST and MHFA programs in the Sámi contexts showed few signs of Sámi-specific adaptations beyond being delivered by Sámi health care personnel or culturally competent non-Sámi personnel. However, one initiative in Norway aimed at producing a northern Sámi version of the SafeTALK intervention, which will be adapted in terms of culture and language.

The identified initiatives were administered by organizations alone, in cooperation or in consortiums. Five initiatives were administered by Sámi and Swedish NGOs, together or alone, and one by a religious organization. Seven initiative were administered by public health care organizations, and the rest by different organizations working together or entering into consortiums. Most initiatives administered by health care organizations were funded by themselves, whereas most other initiatives were funded by a range of publicly available project grants.

While most initiatives lacked thorough evaluation, some process-oriented evaluations in project reports pointed to the importance of anchoring projects locally or being community driven, as well as paying attention to issues of culture and context to make sure projects were appealing and relevant to target populations. One initiative attempted to conduct a questionnaire-based evaluation of the outcomes of providing gatekeeper training workshops, but it was reported that meaningful statistical processing was not possible due to the low response rate.

## Discussion

This study has mapped suicide prevention initiatives targeting Sámi in Norway, Sweden and Finland. The following discussion focuses on some of the characteristics of these initiatives, and our interpretations of what these tell us about how society represents the problem of suicide among Sámi. Also, inspired by the WPR-approach, we asked what problematizations of suicide were not visible in the present study and some implications thereof.

**Fig. 3:**
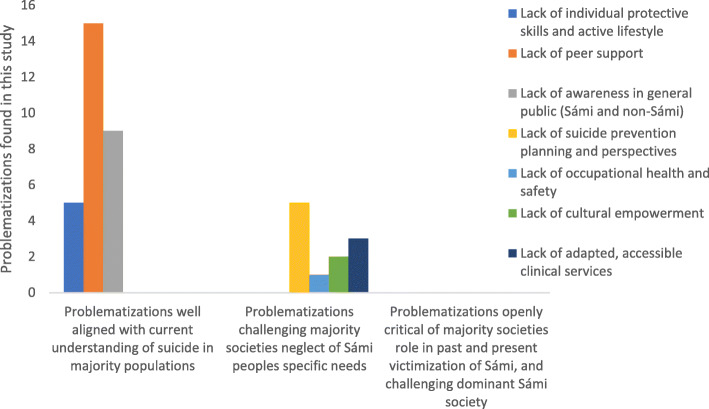
Number of problematizations found in this study, depending on categorization of whether and how such problematizations challenges majority and Sámi societies

Firstly, it should be noted that there was considerable diversity in terms of prevention approaches, as the problematizations and resulting activities sought to understand and address issues on all levels suggested for suicide prevention by the WHO (individual, relational, community/cultural, societal and health systems levels). However, most of the problematizations (three out of four) found in this study placed the problem on individual-relational levels (individual shortcomings or lack of peer support between individuals) or lack of suicide awareness in the general public. Although we regard these focuses and the activities accompanying them as well aligned with national (non-Sámi) prevention policies [15, 16], which are both useful and welcome initiatives in the Sámi context, their dominant position might be problematic for a number of reasons. Firstly, this focus mainly places the problem (and therefore the responsibility to fix it) among struggling Sámi, their peers and the rather unspecific category of “the general public.” This is only partly supported by available evidence. For example, attitudes towards suicide in Sámi risk groups for suicidality was, if anything, less problematic than among non-Sámi peers, reindeer herding Sámi had no different attitudes to suicide as compared to the majority of Swedes [24] and young adult Sámi were actually less prone to think that talking about suicide increases the risk, less likely to think that one could not stop an individual whose mind was made up, and more aware of suicide in general when compared to Swedish peers [25]. Arguably, this points to lack of suicide awareness and peer support not being what’s driving suicidality within Sámi risk-groups for suicidality [24, 25]. Secondly, placing the focus on these particular issues might obscure other important problematizations found in this study, such as the lack of prevention planning and perspectives, shortcomings in health systems, lack of occupational health and safety in reindeer herding and lack of cultural empowerment among Sámi youth. Also, those issues are more aligned with Sámi-specific priorities found in the Sámi suicide prevention plan, which stresses the need to also understand and address suicide through up-stream intermediary factors, including the historical and sociocultural contexts [17]. Actually, when comparing the strategies in the Sámi-specific prevention plan with the problematizations found in this study, we noted that some strategies were not covered at all by the identified initiatives, perhaps because they could be considered critical or challenging towards Sámi or the majority society. For example, we did not identify any prevention rationales that focused on potentially controversial issues such as strengthening of Sámi self-determination, efforts to recognize and deal with historical traumas among Sámi, reducing Sámi exposure to violence (including sexual violence) and ethnic discrimination, and breaking taboo, stigma and negative attitudes related to non-normative sexuality and gender identity among Sámi. What we are suggesting then, is that suicide prevention activities that challenge the status quo in society – be it mainstream majority society or Sámi society – may have been less likely to be initiated and funded (Fig. 3).

The potential consequences of this make-up of problematizations and activities might be that suicide among Sámi, as seen from recent prevention initiatives, is predominantly construed as a problem residing at the individual and relational levels, which also possibly could be solved by raising awareness in the general public. In our view, such an understanding of suicide among Sámi would negate the complexity of the problem and obscure not only the role of factors such as “infringement of native culture and language, lack of territorial, political and economic autonomy, the need for culturally appropriate health systems” [6] but also the need to critically challenge norms within Sámi society that might be contributing to suicide, for example, those associated with masculinity and within-group (“lateral”) violence. However, it should also be noted that activities, such as the truth and reconciliation commission in Norway, the “Queering Sápmi” project in Sweden [26], and the multilevel efforts to address sexual violence in the community of Tysfjord, seem well aligned with strategies in the Sámi prevention plan, although not framed as suicide prevention. This, in turn, raises the critical question if suicide prevention activities really offer the best suicide prevention in this context. For example, Sámi reindeer herders in Sweden have for a long time been pointing to how their culturally grounded industry is negatively impacted by issues like increasing predation of reindeers (due to increased populations of bears, wolves, wolverines, lynxes and eagles), escalating grazing land-conflicts (due to extractive industries, societal infrastructure, tourism development and wind power turbines infringing on the reindeer grazing lands), climate change and outdated legislation. The herders suggest such factors threatens their industry economically and culturally, thus causing mental ill-health and suicidal behaviors among the them. If this is indeed so, can suicide prevention among Sámi reindeer herders be effective without addressing the societal factors driving the deteriorating mental health? At least, it seems the development of the Sámi-specific prevention plan offers future initiatives a path to aligning their priorities with the rationales found in the prevention plan (including focusing on issues like strengthening Sámi self-determination), thus covering a fuller spectrum of activities needed for improved prevention among Sámi.

The most common form of problematization of suicide in this study (lack of peer support) was mainly connected to two different types of initiatives. On the one hand, health care authorities implemented international suicide prevention training programs targeting gate-keepers, the general public or reindeer herding Sámi ASIST in Norwegian and Finnish parts of Sápmi, and MHFA in Swedish parts of Sápmi). On the other hand, there were a smaller group of initiatives targeting Sámi at-risk groups (young men, young reindeer herders, and young reindeer herding men) run by Sámi NGOs. Comparing them is interesting because critical suicide researchers have argued that the first type of program is problematic in Indigenous settings, as they build on assumptions of universality in suicide, suicidal behavior, pedagogic style and conceptualization of distress, which might fail to address the realities of Indigenous participants [27]. Instead Wexler, White [28] propose that suicide prevention in such settings should be built on community-based context-specific approaches, allowing for a better match with Indigenous people’s needs. Actually, the programs run by Sámi NGOs fit that description rather well, as the main content of these initiatives was co-created by the participants themselves, in the form of sharing and discussing suicide from the participants perspectives, and sometimes using Sámi cultural practices (yoiking) as a tool for cultural empowerment. Again, this is well in line with Redvers, Bjerregaard [11], whom after scoping for Indigenous suicide prevention initiatives in the Arctic circumpolar regions from 2004 to 2014, suggested that “there is resounding agreement [among researchers] that culturally-grounded solutions and community-based programs are keys to understanding and approaching suicide prevention.” Building on this, we argue that a discussion on what should be future priorities for Sámi suicide prevention is warranted, not in the least because the initiatives that might be labeled “bottom-up approaches, grounded in community priorities” (the initiatives run by Sámi NGOs) relied on funding from external grants, which was the reason they did not publicly present as suicide prevention activities. However, without proper evaluations, it is difficult to support arguments on whether suicide prevention in Sámi contexts is more appropriate and/or effective using adaptations of universal programs or tailor-made community-based programs.

Concerning the evaluation of initiatives, the main finding was that there was a lack of them. For example, it would have been desirable that initiatives presented clear rationales of how their activities was meant to reduce suicide, that they identified key factors for achieving this, and included predefined measures of success, including outcome measures. The fact that only one initiative tried to measure outcomes using quantitative data suggest that implementers did not try very hard, that they lacked capacity, and/or that the task was too complex. Regardless of the reason, this problem is not unique to this context. Authors of a systematic review of suicide prevention interventions targeting Indigenous peoples in Australia, the United States, Canada and New Zealand succeeded in identifying only nine evaluated Indigenous suicide prevention programs during 1980–2012 [29]. This led them to suggest that evaluation resources and know-how might be unavailable or stretched too thin due to other priorities taking precedence (for both Indigenous communities and research groups), and running scientifically rigorous evaluation programs that are respectful, adapted to cultural and contextual needs, involve Indigenous communities as equal partners and build Indigenous research capacity might simply be to resource demanding. However, Clifford, Doran [29] also highlight that stronger bonds between researchers and communities might lead to less expensive processes. One pragmatic way of addressing this has been developed in Australia, a context with similar characteristics and challenges as Sápmi. There, the “Indigenous Suicide Prevention Activity Evaluation Framework” is an easy-to-use tool being promoted for use by prevention initiative organizers, hopefully resulting in initiatives including evaluation components already at planning stages [30]. From our perspective, and the need to improve the evaluation of suicide prevention initiatives among Sámi, adopting this Australian approach seems both reasonable and feasible.

## Methodological discussion

Although the pragmatic approach chosen to map prevention initiatives was an integral part in making this study possible, the lack of a systematic approach meant taking the risk of knowledge gaps and personal bias influencing the inclusion of initiatives. For example, it is reasonable to assume that personal bias made us more likely to succeed in identifying initiatives we were somehow involved in. Furthermore, excluding identified initiatives when lacking written documentation only if we ourselves had not been involved in them resulted in excluding one initiative, thus confirming that personal bias affected the inclusion/exclusion of initiatives. As a fact, this means that the overview of prevention initiatives targeting Sámi created in this study is not exhaustive.

As is the case in all qualitative research, the specific competencies and experiences of those performing the analysis is reflected through its results. Although we do represent a diverse group of researchers, it is likely that other researchers would have interpreted the same data in at least slightly different ways. However, we sought to increase transparency in describing how analysis was carried out and including supplements with substantial amounts of data regarding each initiative. This makes it possible for the reader to evaluate how reasonable our interpretations were through identifying other potential ways to analyze the same material. In accordance with other qualitative scholars, we argue that this strengthens the overall trustworthiness of the study [31, 32].

## Conclusions

This study identified 17 diverse initiatives specifically targeting Sámi for suicide prevention in Norway (5 initiatives), Sweden [9] and Finland [1], as well as internationally (border-crossing) [2], during 2005–2019. Utilizing the WPR-approach, the analysis of strategies embedded within those initiatives showed that suicide among Sámi was understood to take place because of a perceived lack on multiple levels, all suggested for suicide prevention by the WHO (individual, relational, community/cultural, societal and health systems levels). However, we argue that the dominant perspectives were individual-relational, as well as focusing on informing the general public, which might misplace the problem of suicide among Sámi in the sense that it simplifies the issue and risks obscuring critical approaches, such as including local, Sámi and Indigenous youth perspectives in prevention planning, improving health systems for Sámi, and seeking to promote cultural empowerment among Sámi. Furthermore, the lack of prevention rationales addressing certain issues included in the Sámi-specific prevention plan (lateral violence among Sámi, ethnic discrimination of Sámi, historical trauma inflicted due to colonial practices and exclusion of Sámi with non-conforming sexual or gender identities) led us to question if those have been considered too critical or challenging towards Sámi or majority societies, preventing initiatives from addressing them. However, we acknowledge that the identified findings confirm that there has been considerable effort invested in preventing suicide among Sámi during the last 15 years, and with the development of the Sámi-specific prevention plan (released 2017), future initiatives might potentially align their priorities with its full spectrum of strategies. Also, if the evidence base for suicide prevention among Sámi is to be strengthened and promising practices identified, it is important to improve the evaluation of prevention activities. For this to happen, we suggest that initiative administrators need structural support as regards developing evaluation plans and implementing them.

## Supplementary Information

**Additional file 1: Supplementary Table 1.** Descriptive characteristics of suicide prevention initiatives targeting Sámi in Sweden.

**Additional file 2: Supplementary Table 2.** Descriptive characteristics of suicide prevention initiatives targeting Sámi in Norway, Finland and internationally.

**Additional file 3: Supplementary Table 3.** Problematizations, category and level of intervention suggested, yielded through applying the “What is the problem represented to be?”-approach on suicide prevention initiatives targeting Sámi in Norway, Sweden and Finland.

## Data Availability

Project reports (in Scandinavian and/or English languages) used during the current study are available from the corresponding author on reasonable request.

## Abbreviations

ASIST: Applied Suicide Interventions Skills Training
MHFA: Mental Health First Aid
NGO: Non-governmental organization
WHO: World Health Organization
WPR: “What is the problem represented to be?”
