# Supplementary material for: “Mapping suicide prevention initiatives targeting Indigenous Sámi in Nordic countries”
Source: BMC Public Health. 2021 Nov 7;21:2035. doi: 10.1186/s12889-021-12111-x (PMC8573914; doi:10.1186/s12889-021-12111-x)
Supplement: Supplementary file 1 — Additional file 1: Supplementary Table 1. Descriptive characteristics of suicide prevention initiatives targeting Sámi in Sweden. [file 12889_2021_12111_MOESM1_ESM.docx]

Supplementary table 1. Descriptive characteristics of suicide prevention initiatives targeting Sámi in Sweden.

| **Initiative** | **Samiskt krisnätverk [Swedish: Sámi crisis network]** | **Vaajmoe [south Sámi: heart]** | **Piloting Mental Health First Aid in Västerbotten, Sweden** | **Biegganjunni [north Sámi: whirlwind]** | **Aktavuohta [Lule Sámi: to be in contact with someone]** | **Kunskapsnätverk för samisk hälsa [Swedish: Knowledge network for Sámi health]** | **Mannen myten och Sáminuorra [Swedish: The man the myth and Sáminuorra]** | **Occupational health and safety plans in reindeer herding communities in Sweden** | **Folkmöte om suicidproblematik [Swedish: Public meeting on suicidality]** |
| --- | --- | --- | --- | --- | --- | --- | --- | --- | --- |
| **Program administrator** | Swedish church | Sáminuorra (Sámi youth association in Sweden) | Socialpsykiatrisk centrum, Region Västerbotten | Sámiid Riikasearvi (The National Association for Sámi in Sweden) | Sámiid Riikasearvi (The National Association for Sámi in Sweden) | Region Norrbotten, Region Västerbotten, Region Jämtland och Härjedalen, Region Dalarna in collaboration with Sámi Norwegian National Advisory Unit on Mental Health and Substance Abuse (SANKS), the Sámi parliament in Sweden and Sámi NGOs in Sweden. | MÄN (a Swedish NGO for men to act against men’s violence towards women) in collaboration with Sáminuorra (Sámi youth association in Sweden) | Sámiid Riikasearvi (The National Association for Sámi in Sweden) | SANKS, Norrskenet primary health care centre, Swedish church (Vittangi congregation), Knowledge network for Sámi health, and Sohppar Sámisearvi (Association for Sámi in Soppero) |
| **Source of information** | Project reports (2) | Project report, first-hand account. | Project report, emails with organizers. | Project report, first-hand account. | Project report, first-hand account. | Project report, phone calls and emails with organizers. | First-hand account | Project report. | First-hand account |
| **Year(s)** | 2007-2017 | 2010-2015 | 2012 | 2012-2013 | 2014-2015 | 2017- | 2018- | 2018- | 2019 |
| **Country** | Sweden | Sweden | Sweden | Sweden | Sweden | Sweden | Sweden | Sweden | Sweden |
| **Target group for suicide prevention** | At-risk Sámi in Sweden | Sámi youth in Sweden | Sámi reindeer herding community members in Västerbotten county, Sweden. | Young Sámi reindeer herding men in Sweden (17-35 years of age) | Young Sámi reindeer herders in Sweden (18-25 years of age) | Sámi health care personnel and providers in Swedish Sápmi. Sámi in Norrbotten, Västerbotten, Jämtland and Härhedalen, and Dalarna counties. | Young Sámi men in Sweden (16-30 years of age) | Members of Sámi reindeer herding community of Raedtevaerie, Sámi in Sweden (leaflet) | The Sámi population of upper Torne river valley, in Sweden |
| **Project aim/ mission** | Deliver easy access to crisis support by Sámi volunteers. | Yoik and sing for cultural empowerment and supporting each other, as well as to raise awareness of mental health and suicidality among Sámi youth | Pilot the Mental Health First Aid (MHFA) course in Sweden, train 2000 individuals and evaluate the program. Within this program, to train Sámi gate keepers in the Mental Health First Aid program. | Arrange inspirational workshops for young reindeer herding men, promoting gender equality. Strengthen peer support through sharing and talking about life experiences. Strengthen mental well-being through teaching conflict management skills. | Spread information about life as a young reindeer herder. Create a network for young Sámi reindeer herders, arrange educational workshops for them, including to share survivors’ stories, educate in conflict negotiation techniques and mental health promotion. | Strengthening health care practitioner’s knowledge in Sámi health to improve quality and access for Sámi patients. To train Sámi health personnel to be MHFA instructors and train Sámi in the program. | Challenge destructive masculinity in a Sámi context, including to strengthen social support between young men, culturally empower them through arranging a yoik workshop and train them to use yoik as a tool for emotional regulation. | Supporting a Sámi reindeer herding community (RHC) to create and implement an occupational health and safety plan as a pilot. Using the experience of the pilot to support other RHCs to do the same. Producing a leaflet on suicide prevention among Sámi to be distributed to Sámi in Sweden. | Raise awareness about suicide and train the public in the SafeTALK program |
| **Delivery methods including participants, if available** | Telephone service manned by Sámi volunteers on Friday and Sunday nights | Three workshops, three recording sessions for a CD recording. An unknown number of less organized gatherings | One two-day MHFA course was carried out with participants from one Sámi reindeer herding community. | Three two-day workshops focused on strengthening peer support and conflict management | Nine women and 11 men participated in six two-day workshops (of which one primarily focused on promoting psychosocial well-being), a Facebook group for in-group communication, and an Instagram account for spread of information to the general public. | Creating a network for knowledge transfer among health care practitioners in the regional health care authorities in Sápmi (on the Swedish side). Developing a digital Internet-based tool for providing cultural training for health care personnel in Sámi areas. Seven Sámi health workers were trained to be MHFA instructors. Three two-day MHFA courses were carried out. | About 10 men participated in one yoik workshop focused on exploring yoik (traditional singing) as a tool for emotional regulation. | A consultant conducted interviews and group consultations used for creating an occupational health and safety plan. A leaflet with articles on suicide awareness was distributed along with monthly Sámi media “Samefolket”. | A public meeting on suicidality and a half-day SafeTALK workshop in Övre Soppero, Norrbotten county (40 persons). |
| **Evaluation (main content)** | The hotline was used 52 times during 2007 to 2010 and one time between 2015 and mid-2016. No measures in between. Low usage rate was attributed to low professionalism in service deliverance (no supervision for volunteers). The service was shut-down due to low usage of service | Project report includes some process evaluation, in which project coordinators report participants growing personally and as a group. It is also mentioned that the project generated media attention, thus contributing to raising awareness | The national project included a randomized controlled trial and a focus group study, but no analysis was conducted on delivering MHFA to Sámi specifically. However, one planned MHFA course with members from different reindeer herding communities was canceled due to low turnout of participants. | Project report includes some process evaluation, stating that activities were carried out as planned and a majority of participants reporting having enjoyed taking part in the project | Project report includes process evaluation, which included reports of participants being very positive to having taken part in the project, some describing it as “life changing.” Generally, the project was carried out according to plan, with exception of one workshop (out of six) where several facilitators fell ill at the same time | Process evaluation suggests difficulties in recruiting participants for MHFA courses. In improving this Region Jämtland Härjedalen will be developing a new program based on mental health literacy training combined with traditional Sámi handicraft (Vætnoe) | On-going project. The planned workshop was postponed due to too few planning to attend. Higher turnout (about 10 participants) was achieved after partnering with a Sámi education institute | No evaluation was performed, but fewer activities than planned were carried out due to long start-up, which was related to a great need to inform the consultant of the Sámi reindeer herding culture and context | Not available |
