# Supplementary material for: “Mapping suicide prevention initiatives targeting Indigenous Sámi in Nordic countries”
Source: BMC Public Health. 2021 Nov 7;21:2035. doi: 10.1186/s12889-021-12111-x (PMC8573914; doi:10.1186/s12889-021-12111-x)
Supplement: Supplementary file 2 — Additional file 2: Supplementary Table 2. Descriptive characteristics of suicide prevention initiatives targeting Sámi in Norway, Finland and internationally. [file 12889_2021_12111_MOESM2_ESM.docx]

Supplementary table 2. Descriptive characteristics of suicide prevention initiatives targeting Sámi in Norway, Finland and internationally.

| **Initiative** | **Samisk psykiatrisk ungdomsteam (PUT) [Norwegian: Sámi Psychiatric Youth team]** | **Åpenhet og nærhet,(Norwegian: Openness and Intimacy)** | **Public meetings and ASIST in South Sámi areas** | **Finnmark – et selvmordstryggere samfunn [Norwegian: Finnmark – a suicide safe society]** | **Adaptation of SafeTALK to Northern Sámi** | **EALLIN [North Sámi: Life]** | **Selvmordsforebygging i Sápmi / Preventing suicide in Sápmi** | **Circumpolar Resilience, Engagement and Action Through Story (CREATeS)** |
| --- | --- | --- | --- | --- | --- | --- | --- | --- |
| **Initiative administrator** | Sami Norwegian National Advisory Unit on Mental Health and Substance Abuse (SANKS), Finnmark hospital trust, Norway. | A local consortium led by the municipality (health and social services and the school), including local associations, Leve Finnmark and Mental Helse, and Sami Norwegian National Advisory Unit on Mental Health and Substance Abuse (SANKS). | Sami Norwegian National Advisory Unit on Mental Health and Substance Abuse (SANKS) | County governor of Finnmark, Northern Norway Violence, Traumatic Stress and Suicide Prevention Resource Centre (RVTS Nord), Sami Norwegian National Advisory Unit on Mental Health and Substance Abuse (SANKS) | VIVAT Selvmordsforebygging in cooperation with the Regional resource centres for Violence, Traumatic Stress and Suicide Prevention in Northern and Mid Norway (RVTS Nord and RVTS Midt) and SANKS | Utsjok primary Health care centre | Sami Norwegian National Advisory Unit on Mental Health and Substance Abuse (SANKS) and Saami Council | Inuit Circumpolar Council (ICC), Finland and Canada, under the Arctic Council Sustainable Development Working Group (SDWG). |
| **Source of information** | Service description in other report. First-hand account. | Project report. First-hand account. | First-hand account. | Project report, first-hand account. | First-hand account. | First-hand account. | Project report (prevention plan). First-hand account. | Project report |
| **Year** | 1990-2020 | 2007-2008 | 2010 - 2011 | 2013-2015 | 2018 - | 2017-2018 | 2015-2017 | 2018-2019 |
| **Country** | Norway | Norway | Norway | Norway | Norway | Finland | International | International |
| **Project aim/ mission** | Deliver easy access psychiatric treatment of suicidality and drug use, which meets the needs of culturally and language competent care for young Sámi | Implementation of a multilevel approach to suicide prevention among youth and adults in Tana municipality, including training gate keepers in the Applied Suicide Intervention Skills Training (ASIST) program, reduce vulnerability to suicide and strengthen resilience in youth by early recognition and referral for mental health issues, supporting an active lifestyle for identified at-risk youth, mental health literacy training in school, strengthen peer support through creating meeting places for well-being. | Raise awareness about suicide and train gate keepers in the Applied Suicide Intervention Skills Training (ASIST) program | Raise awareness about suicide and train gate keepers in the Applied Suicide Intervention Skills Training (ASIST) program | Develop a northern Sámi adaptation of the SafeTALK program, adapted to Sámi culture and available in Northern Sámi language. | Train gatekeepers in the Applied Suicide Intervention Skills Training (ASIST) program | Create a plan for suicide prevention among Sámi in Norway, Sweden and Finland. | To create stories for youth engagement and knowledge translation to support the suicide prevention and mental wellness efforts of the Arctic States. Create and share digital stories related to suicide from the perspective of Arctic Indigenous youth |
| **Target group for suicide prevention** | At-risk young population (15-30 years of age) in Sámi core areas in Northern Norway, and Sámi throughout Norway (some service delivery in Sweden via distance bridging technology). | The general and at-risk youth and adult population of Tana municipality (Sámi and non-Sámi), Norway. | The Sámi population in south Sámi areas in Norway. | The general population of Finnmark county, including the population of Sámi core areas in Norway. | Sámi in Norway. | Sámi in Finland. | Sámi in Norway, Sweden and Finland | Arctic Indigenous youth. Sámi youth from Norway, Sweden and Finland (18-25 years of age). |
| **Delivery methods including participants, if available** | Psychiatric outpatient services in person and/or with distance bridging technology. | Five ASIST courses, with 85 participants (20% men). Mental health literacy training in school for older kids, “Zippy’s friends” curriculum for young kids (focusing on developing coping skills), and information meetings at the youth club. Screening of mental health in 9th grade and follow-up talks with school nurse for referral. Outdoor fun physical activities (canoeing, dog-sledding, snow-boarding, kiting, rock climbing, etc.) for specifically targeted youth (13-25 years of age). Information meetings regarding suicide and mental health with local village associations. Set up of local association for bereaved. Local events on international suicide prevention days. Creation of Tana municipality plan for suicide prevention (including most activities listed above). | An unknown number of public meetings on suicide prevention, combined with an unknown number of ASIST courses. | Ten public meetings on suicide prevention, combined with 13 ASIST courses, in nine of the municipalities in Finnmark, including in Sámi core areas (Kautokeino, Karasjok, and Tana/Nesseby). | Development of Northern Sámi adapted SafeTALK program. | Six ASIST courses delivered in northern Finland. | Reviewing literature on suicide and suicidality among Sámi in Norway, Sweden and Finland. Conducting consultations with Sámi grassroots involved in suicide prevention and developing a plan for suicide prevention based on available literature and consultations. Plan released March 2017. | Six Sámi participated in two digital storytelling workshops (one in Finland) and one knowledge translation workshop. |
| **Evaluation (main content)** | According to a report from 2015 (not updated), the service serves 80-120 patients per year, and no patients have died by suicide while being a patient (no follow-up). Service discontinued in 2020. | Project report includes process evaluation, which showed most planned activities were carried out according to plan, while recruiting men to ASIST courses was difficult, even with approx. 80 specifically selected men being invited. Success factors are reported to have been: local leadership, high sense of urgency and commitment from municipality and local organizations, and support from outside professionals (SANKS and RVTS-Nord). | Not available | Evaluation showed that 298 persons (unknown ethnicity) took the ASIST courses, and t the project appeared in 17 media pieces, of which six where in Sámi media. A questionnaire given to participants got too few responses for meaningful statistical evaluation and was hence not reported. | Not available (on-going project) | Not available | Not available | Project report includes process evaluation, which showed activities had been carried out according to plan, and participating youth reporting finding it challenging, meaningful, positive and enjoyable to take part in the project. |
