# Supplementary material for: “Mapping suicide prevention initiatives targeting Indigenous Sámi in Nordic countries”
Source: BMC Public Health. 2021 Nov 7;21:2035. doi: 10.1186/s12889-021-12111-x (PMC8573914; doi:10.1186/s12889-021-12111-x)
Supplement: Supplementary file 3 — Additional file 3: Supplementary Table 3. Problematizations, category and level of intervention suggested, yielded through applying the “What is the problem represented to be?”-approach on suicide prevention initiatives targeting Sámi in Norway, Sweden and Finland. [file 12889_2021_12111_MOESM3_ESM.docx]

Supplementary table 3. Problematizations, category and level of intervention suggested, yielded through applying the "What is the problem represented to be?"-approach on suicide prevention initiatives targeting Sámi in Norway, Sweden and Finland.

| Problematizations | Category | Level |
| --- | --- | --- |
| Young Sámi men do not have enough tools for emotional regulation | Lack of individual protective skills and active lifestyle | **Individual** |
| Sámi (and non-Sámi) youth at risk of suicidality do not have an active enough lifestyle |  |  |
| Sámi (and non-Sámi) young kids do not have enough coping skills to deal with life's challenges, conflicts and mental health issues |  |  |
| Young reindeer herders do not have good enough skills to take care of themselves/increase mental well-being |  |  |
| Young male reindeer herders do not have good enough conflict management skills |  |  |
| Sámi youth do not have enough access to peer-support | Lack of peer support | **Relational** |
| Young male reindeer herders do not have enough access to peer support |  |  |
| Young reindeer herders do not have enough access to peer support |  |  |
| Young Sámi men do not have enough access to peer support |  |  |
| At-risk Sámi do not have enough access to adapted crisis support (telephone helpline run by Sámi volunteers) |  |  |
| Reindeer herding Sámi do not have enough knowledge to recognize individuals with a mental health and substance use-related crisis and to intervene, including referring to professionals |  |  |
| Sámi do not have enough knowledge to recognize individuals with a mental health and substance use-related crisis and to intervene, including referring to professionals |  |  |
| Sámi do not have enough knowledge to recognize when someone is thinking about suicide and connect them to an intervention provider |  |  |
| Sámi and non-Sámi do not have enough knowledge and skills to recognize persons at-risk of suicide and to intervene, including referring to professionals |  |  |
| Sámi do not have enough knowledge and skills to recognize persons at-risk of suicide and to intervene, including referring to professionals |  |  |
| Sámi do not have enough knowledge and skills to recognize persons at-risk of suicide and to intervene, including referring to professionals |  |  |
| Sámi needs culturally and language-wise adapted course curriculum to recognize signs of suicidality, engaging individuals in talking about it, and connecting them to an intervention resource |  |  |
| Sámi (and non-Sámi) do not have enough knowledge and skills to recognize persons at-risk of suicide and to intervene, including referring to professionals |  |  |
| Sámi (and non-Sámi) school kids do not have enough knowledge regarding how to support each other for improved mental health |  |  |
| Sámi (and non-Sámi) bereaved by suicide do not have enough peer support |  |  |
| Sámi reindeer herding communities do not work enough on occupational health and safety | Lack of occupational health and safety | **Community/Culture** |
| Sámi youth are not culturally empowered enough | Lack of cultural empowerment |  |
| Young Sámi men are not culturally empowered enough |  |  |
| Indigenous youth are not enough included in knowledge translation regarding suicide prevention in the Arctic | Lack of suicide prevention planning and perspectives | **Societal**  **Societal** |
| Sámi reindeer herding communities are not structured enough in their occupational health and safety efforts |  |  |
| Suicide prevention for Sámi (and non-Sámi) is not enough strategized on the local level |  |  |
| Sámi-specific suicide prevention needs are not addressed by existing general (universal) suicide prevention work |  |  |
| Suicide prevention among Sámi is not strategized enough for effective prevention |  |  |
| The general public is not enough aware enough of mental health and suicide among Sámi youth | Lack of awareness in general public (Sámi and non-Sámi) |  |
| The general public is not aware enough of the lives of young reindeer herders |  |  |
| The general public is not aware enough of how Indigenous youth understand suicide in the Arctic |  |  |
| Sámi are not aware enough of the issue of suicide among them |  |  |
| Sámi and non-Sámi are not aware enough of the issue of suicide among them |  |  |
| Sámi are not aware enough of the issue of suicide among them |  |  |
| Sámi public are not aware enough of suicide among them |  |  |
| Sámi (and non-Sámi) are not aware enough of suicide among them |  |  |
| The general public is not aware enough of the issue of suicide among Sámi |  |  |
| Young Sámi at-risk population do not have enough access to Sámi-specific (culturally and language adapted) psychiatric treatment of suicidality and drug abuse | Lack of adapted, accessible clinical services | **Health systems** |
| Health care personnel are not knowledgeable enough in regards to Sámi health, to be able to deliver good enough quality and access to care to Sámi patients |  |  |
| Sámi (and non-Sámi) youth at-risk of mental health issues are not recognized and referred to psychiatric services to a large enough extent by school health services |  |  |
